# The anatomy of rage: conceptualization, operationalization, and validation of a new measurement scale

**DOI:** 10.3389/fpsyt.2025.1648903

**Published:** 2025-11-17

**Authors:** Natalia Michałkiewicz, Paweł Strojny, Agnieszka Strojny

**Affiliations:** 1 Doctoral School in the Social Sciences, Jagiellonian University, Kraków, Poland; 2 Institute of Applied Psychology, Faculty of Management and Social Communication, Jagiellonian University, Kraków, Poland

**Keywords:** rage, tilt, gaming disorder, emotional dysregulation, digital game

## Abstract

**Introduction:**

Despite its prevalence among gamers, rage remains an understudied phenomenon in digital gaming. The present study aimed to conceptualize rage as an emotional response to in-game failure or frustration and to develop a reliable and valid self-report instrument to measure this experience.

**Methods:**

The study was conducted in two phases. In the first, theoretical and qualitative phase, a focus group and expert assessment procedure were used to define the construct of rage in gaming and develop initial items. In the second, empirical phase, an international sample of 267 participants (126 females, 124 males, 17 identifying as other; aged 18–52) completed an online survey. Exploratory and confirmatory factor analyses were conducted alongside reliability testing and correlational analyses with related psychological constructs (DERS-SF, IGDS9-SF, BAQ, PHQ-9, GAD-7).

**Results:**

Factor analyses resulted in an 11-item scale with a three-factor structure: Emotion, Cognition, and Arousal. The scale demonstrated good psychometric properties, including satisfactory reliability and model fit. Correlations with related constructs indicated significant associations between rage, gaming disorder symptoms, and emotional dysregulation, suggesting that gaming rage is linked to maladaptive emotional and behavioral patterns and warrants further investigation.

**Conclusion:**

The developed scale offers a preliminary tool for future research on rage in video game players. Its application may contribute to a more comprehensive understanding of this phenomenon and its psychological correlates.

## Introduction

Since the days of shooting pixelated spaceships, the gaming industry has grown into a $187.7 billion sector (1). The rapid development of game design, fueled by advanced technological capabilities, has significantly enhanced player immersion and engagement. These advancements have not only elevated gameplay experiences but also deepened players’ emotional involvement. A relatively new and still underexplored phenomenon in the context of players’ emotional experiences is rage (also referred to as tilt).

The terms rage and tilt are commonly used by players to describe intense emotional reactions during gameplay and have emerged organically within gaming communities. The term tilt was first used by poker players to describe a state of emotional disruption leading to loss of control over behavior, often evident in altered decision-making and reduced strategic focus (2, 3) Tilt is associated with negative experiences such as disappointment, anxiety, depressive thoughts, and sleep disturbances (4), and has also been identified as a potential risk factor for gambling addiction (5). It is typically defined as a shift in a player’s behavior (relative to their standard style of play), most often triggered by ‘bad gambling’ (e.g., losing despite having a statistically advantageous hand) or ‘needling’ (deliberate provocations by other players to irritate or offend), and is strongly linked to feelings of frustration (2, 5).

In video gaming, the situation is less clear. Both rage and tilt are terms frequently used by players, content creators (e.g., YouTube compilations of gamers rage), and in player-driven forums such as Reddit to describe intense emotional outbursts during gameplay (6–8). Despite the widespread presence of the phenomenon, the terminology is rarely defined explicitly and is used inconsistently across studies and platforms. Some studies use rage as the primary label, yet intermittently refer to tilt as if it were synonymous, without clearly distinguishing between the two (9, 10). Other papers focus exclusively on one term while providing definitions that conceptually overlap with the other construct (11). Still others use tilt as the central term, yet offer diverging definitions that highlight different facets of the phenomenon. For example, Bonilla et al. (12) describe tilt primarily as a pattern of escalating behavior driven by repeated failures in performance-based tasks, which then gives rise to frustration and functional impairment. In contrast, Wu et al. (13) define tilt as an internal emotional state triggered by external stressors (e.g., poor gameplay or provocation), characterized by reduced cognitive control, negative affect, and compromised decision-making. This fragmented use of terminology, across and even within studies, underscores the lack of conceptual clarity and the need for terminological standardization in this area of research.

Our aim is not only to explore the psychological foundations of the phenomenon but also to help clarify and standardize the language surrounding it. In this paper, we use the term rage to describe an intense emotional reaction that influences the player’s behavior during gameplay. While rage and tilt are often used interchangeably, we chose rage due to its stronger emotional connotation, which aligns with the affective focus of our study and the purpose of our scale. Moreover, tilt tends to appear more frequently in the context of professional players, with an emphasis on performance decline, which was not addressed in our research (12, 14, 15). The conceptualization of rage used in this paper will be introduced in the next sections. We also acknowledge that terminology may vary across subcultures, games, and platforms, and players may interpret or apply these terms differently depending on context and experience.

Although rage is often conceptualized as a basic or primary emotion characterized by intense anger and physiological arousal (16), gaming rage refers to a distinct, situational phenomenon tied to interactive digital contexts. It emerges specifically from in-game failures, perceived lack of control, technical issues, or team-based frustrations, and is marked by affective dysregulation and impulsive reactions such as verbal outbursts or rage-quitting (10). Qualitative investigations further reveal that gaming rage disrupts cognitive processes, such as attention, and is socially reinforced within competitive environments (9). Thus, gaming rage cannot be equated with general anger; it represents a contextualized emotional episode rooted in game-specific triggers, expectations, and mechanics.

Findings from qualitative research (9, 10, 13) suggest that during episodes of rage, players most frequently engage in verbal aggression, such as insulting or attacking other players or the game itself with occasional manifestations of physical aggression (e.g., hitting the desk or throwing a mouse) or abruptly leaving the game (rage quitting). Players report several consequences of such episodes, including impaired in-game performance, emotional “contamination” of other players, and a diminished interest in gaming altogether. Some of these behaviors, particularly those directed toward other players, may be considered as toxic within the gaming environment (17), which has been shown to adversely affect both victims and perpetrators. Individuals repeatedly exposed to in-game aggression exhibit higher levels of depressive symptoms and problematic gaming, while those who act as both perpetrators and victims show elevated levels of anxiety and anger rumination (18).

Rage has also been linked to broader mental health problems, including depression and anxiety (18) as well as gaming disorder (GD), which is characterized by impaired control over gaming and persistence despite negative consequences. A meta-analysis of 61 studies estimated the prevalence of GD to be approximately 3.3% (19). Research indicates that gaming disorder negatively impacts multiple dimensions of health, contributing to physical problems, heightened anxiety and depression, behavioral difficulties, and impaired social functioning (20–22). Given this, examining rage may provide important insights into emotional mechanisms that contribute to the onset and maintenance of problematic gaming.

Even though rage is not formally recognized as a symptom of emotional dysregulation, the high intensity and recurrence of such episodes may reflect difficulties in employing functional regulatory strategies. Emotional dysregulation is associated with maladaptive strategies, such as emotional suppression, which hinder appropriate responses and flexible adjustment to situational demands (23). It has also been recognized as a transdiagnostic factor underlying a wide range of mental disorders (24). Difficulties in emotion regulation have been linked to conditions including depression and generalized anxiety disorder (25), borderline personality disorder (26), autism spectrum disorder and ADHD (27), as well as post-traumatic stress disorder. Findings from a recent meta-analysis indicate that emotional dysregulation is strongly associated with all forms of addiction – both substance-related and behavioral (28). Rage can also be linked to impaired impulse control and difficulties in maintaining goal-directed behavior in the face of negative emotions, both key indicators of emotion dysregulation (29). Hypothetically, it may serve as an outlet for emotions that remain unacknowledged or unexpressed throughout the day, with gaming providing a socially and contextually permissible space to release emotional tension, functioning as a form of emotional acting out in a contained virtual environment.

Given its close links with gaming disorder and emotion dysregulation, rage should be systematically studied as a clinically relevant phenomenon. However, despite its prevalence and relevance, rage in gaming contexts has not yet been examined with instruments that directly capture its emotional foundations. The only available tool, the TILT questionnaire (12), focuses primarily on the perceived causes and consequences of rage episodes, with items such as “I have made the wrong decision” or “I have felt that the game was not fair”. While this represents a valuable contribution, its conceptualization centers on external manifestations and gameplay-related factors rather than the underlying emotional processes emphasized in both theoretical frameworks and empirical research on rage and tilt (2, 5, 9, 13). In contrast, the Rage in Gaming scale (RIG) was developed to assess the emotional core of rage, grounded in the General Aggression Model (30), providing a more theory-driven and comprehensive perspective. Put together, the two instruments can be viewed as complementary: TILT reflects situational and behavioral correlates, whereas RIG provides insight into the affective and cognitive mechanisms that drive rage. By focusing on these emotional foundations, the RIG may advance both theoretical understanding and clinical assessment of rage in gaming contexts.

This study has three main objectives:

- To develop a conceptual definition of rage;
- To construct and validate a novel instrument for measuring rage as an emotional response;
- To explore the relationships between rage, gaming disorder, and emotional dysregulation.

## 2 Materials and methods

The study was conducted in two phases, combining qualitative and quantitative methodologies. In the first phase, a qualitative focus group interview was conducted with four participants in the form of a semi-structured interview to gather insights into the phenomenon of rage in gaming. Based on the thematic analysis of the focus group discussion, an initial pool of items was generated. These items were subsequently evaluated by a panel of expert to assess content validity and clarity.

In the second, quantitative phase, an online survey was administered. Data collected from this phase was used to perform an exploratory and confirmatory factor analysis of the newly developed questionnaire.

### Phase 1: conceptualization and development of the RIG scale

#### Theoretical framework

The theoretical foundation for the development of the questionnaire was the General Aggression Model (GAM) (30), an integrated framework that combines multiple perspectives to explain how aggressive behavior emerges and has been extensively validated in empirical research (31–33). Unlike basic emotion theories or emotion regulation models, GAM provides a broader account that extends beyond emotional processes to incorporate situational, cognitive, social, and personality factors, which makes it particularly suitable for understanding rage in gaming contexts. Rage is conceptualized in this study as an affective reaction, yet one that does not occur in isolation; it is shaped by the interplay of predispositional factors, situational triggers, and the individual’s regulatory capacities.

According to GAM, aggressive behavior arises from the interaction of three core components: input factors (including biological, personality, and social variables), internal states (cognition, arousal, and affect), and decision processes (reflective or automatic) (30). On a distal level, the model also emphasizes the role of experiences and learning processes in developing aggression-related tendencies over time (30).

In the context of gaming rage, input factors may include trait-level characteristics such as impulsivity or prior exposure to permissive attitudes toward aggression. Internal states were operationalized in this study through three dimensions: Arousal, Cognition, and Emotion (the latter chosen over Affect due to conceptual ambiguity in the literature, where Affect sometimes subsumes arousal (34)). Finally, decision processes determine whether these internal states translate into aggressive behavior; for example, players with stronger emotion regulation abilities may inhibit escalation, whereas others may resort to verbal aggression or rage-quitting.

In digital gaming environments, GAM offers a particular advantage by capturing the dynamic interplay between personal predispositions, in-game stressors, and real-time emotional responses. Its explanatory power lies in linking micro-level processes of cognition, arousal, and emotion with broader behavioral outcomes, making it a strong framework for modeling rage in interactive contexts.

#### The definition of rage

The next step involved developing a definition of the phenomenon, which so far has only been precisely defined once in the existing literature. Bonilla et al. (12) described tilt as behavior that triggers frustration, which in turn may lead to anger, reduced attention, and decreased performance. Although their description corresponds closely to what is commonly understood as rage, it was not adopted in the present study due to its lack of alignment with the theoretical model applied, as well as findings from previous research indicating that a player’s behavior is a consequence of tilt rather than its cause (9, 13). Nevertheless, prior studies highlight the important role of behavioral components in understanding this phenomenon (9).

Based on the General Aggression Model (30), previous research findings (9, 13), and results from an original focus group study (discussed later in the text), the following definition was developed: rage is an escalating, aversive emotional reaction, often associated with feelings of anger and irritation, experienced by individuals playing video games in response to failures such as losing, game malfunctions, or the behavior of other participants (including both human players and computer agents). Rage encompasses three aspects: emotion, arousal, and cognitions influenced by individual and situational factors. In some cases, it may lead to aggressive behaviors such as using profanity or throwing objects. The definition was positively evaluated by four experts: three PhD-level psychologists and one professor. Two experts specialized in emotion psychology, while the other two focused on gaming psychology, which ensured both theoretical and domain-specific perspectives were considered in the evaluation.

#### Item development

The initial phase involved a focus group conducted in March 2024 to explore the emotional and experiential dimensions of gaming-related rage and inform scale development. Participants were recruited via purposive sampling from local and gaming-related online communities to ensure diversity in rage intensity and game preferences. Inclusion criteria required adult regular gamers who reported experiencing in-game rage to varying degrees. During recruitment, participants provided age, occupational status, rage intensity, preferred genres, and gaming motivation. Four individuals (two women aged 22, 23; two men aged 28, 44) participated in a two-hour, in-person session. All participants provided informed consent and did not receive any compensation. The focus group followed a semi-structured format with a prepared topic guide, covering: (a) the definition and subjective understanding of rage, (b) emotional and physical symptoms, (c) triggers and contextual causes, (d) perceived consequences, (e) coping strategies, and (f) genre-related variation. The discussion remained flexible to allow for emergent, participant-driven content. The session was recorded, transcribed, and analyzed using six-step thematic analysis (35), with a semantic, inductive approach. The first author manually coded the transcript, identified patterns, and organized them into themes. These reflected multiple dimensions of gaming-related rage, such as emotional escalation, loss of control, guilt, frustration, behavioral expressions, situational triggers, social dynamics, and coping strategies, among other themes identified in the analysis. Although data saturation was not formally assessed, thematic convergence was observed across participants. To deepen understanding, **Supplementary Materials** include anonymized quotes and brief participant descriptions.

Based on the data collected, the similarity of the phenomenon to tilt in gambling, and previous research (2, 9, 10, 12, 13) an initial pool of 40 questionnaire items was created, aiming to capture the frequency of deviations from players’ typical gaming patterns that may indicate the occurrence of rage. A detailed item-tracking table, including all 40 original items with expert ratings, factor loadings, and reasons for deletion or retention, is provided in the **Supplementary Materials**.

#### Content validity

The items were evaluated by the aforementioned experts, whose task was to assess the content validity of each item in relation to the presented definition of the phenomenon (on a scale from 1 – *completely inaccurate* to 5 – *highly accurate*), as well as to assign each item to the appropriate subscale (Emotion, Arousal, Cognition). The experts were also given the opportunity to provide comments on individual items and on the scale as a whole. Based on the following criteria: the absence of any rating of 1 (*completely inaccurate*) by any expert, high accuracy ratings, consistency in subscale assignment between experts, and alignment with the definition of the phenomenon, 22 items were excluded. Additionally, the item *“*I make impulsive decisions*”* turned out to be problematic, it was classified as fitting both the Emotion and Cognition subscales. There was also some concern that impulsive decision-making might be better understood as a behavioral manifestation, an effect of rage. However, due to its conceptual relevance to the definition of the phenomenon, this item was retained, as final decisions were guided not only by statistical criteria but also by theoretical alignment with the construct of rage. As a result, a final list of 18 items was retained, which is presented in **Table 1**.

**Table 1:** Preliminary item set of the Rage in Gaming Scale (RIG) used in EFA and CFA.

Subscale	Item number	Item content
Emotion	RIG_1	I feel annoyed
	RIG_2	I feel irritated
	RIG_3	I feel angry
	RIG_4	I feel frustrated
	RIG_5	I feel embittered
	RIG_6	I experience mood swings
	RIG_7	I get upset more easily
Arousal	RIG_8	I feel tension in my body
	RIG_9	I feel as if my heart is beating faster
	RIG_10	I notice involuntary bodily reactions (e.g., changes in breathing, sweating, hand shaking, or trembling
	RIG_11	I feel stimulated
Cognition	RIG_12	I make impulsive decision
	RIG_13	I feel powerless
	RIG_14	I feel helpless
	RIG_15	I feel disappointed
	RIG_16	I lose focus in the game
	RIG_17	I feel confused
	RIG_18	It’s harder for me to think

### Phase 2: psychometric evaluation

#### Participants

The study was conducted online between March and April 2025 using the Qualtrics survey platform (https://qualtrics.com/). The survey link was distributed internationally via gaming-related groups on Facebook and Discord. Participation required informed consent, being at least 18 years old, playing video games for at least one hour per week, and correctly answering two attention check questions. Out of the 286 individuals who completed the entire study, 18 provided at least one incorrect response to an attention check, and 1 participant was excluded from the analysis as an outlier due to reporting an implausible average of 24 hours of gaming per day (i.e., >3 standard deviations from the mean). As a result, the final sample for analysis comprised 267 participants. Of these, 126 identified as female (M = 24.79 years, SD = 6.52, range = 18–50), 124 identified as male (M = 26.09 years, SD = 6.82, range = 18–52), and 17 identified as other (M = 24.11 years, SD = 5.41, range = 18–38). Participants did not receive any compensation for taking part in the study. According to the guidelines of the authors’ institution and relevant Polish regulations, this study did not require approval from an ethics committee. The procedure was non-invasive, anonymous, and posed no risk of harm to the participants. The study was conducted in line with the ethical principles outlined in the Declaration of Helsinki and complied with the General Data Protection Regulation (GDPR) standards.

#### Measures

##### Rage in gaming

Rage in Gaming (RIG) is an 11-item self-report instrument designed to assess the tendency to experience rage during gaming. It comprises three subscales: Emotion, Cognition, and Arousal. Drawing on previous research in the domain of gaming-related rage, where rage has been conceptualized as a deviation from one’s typical gaming patterns, the scale aims to capture this shift. Respondents indicate how frequently they experience a given state compared to their usual way of playing. Responses are rated on a 5-point Likert scale ranging from 1 (*Never – I do not notice such a change*) to 5 (*Very often – I notice such a change*). The total score ranges from 11 to 55, with higher scores indicating a greater tendency toward rage in gaming. The internal consistency of the overall scale was high, with Cronbach’s alpha of .87.

##### Difficulties in emotion regulation scale-SF

Difficulties in Emotion Regulation Scale (DERS-SF) (36) is an 18-item self-report instrument assessing six domains of emotion dysregulation. Items are rated on a 5-point Likert scale (1 = *Almost Never*, 5 = *Almost Always*). The scale has demonstrated strong psychometric properties, with Cronbach’s α ranging from .89 to .91 in prior research (36). In the present study, internal consistency was α = .89.

##### Internet gaming disorder scale–short form

The Internet Gaming Disorder Scale – Short Form (IGDS9-SF) (37) is a 9-item self-report instrument assessing Internet Gaming Disorder based on DSM-5 criteria (38). Items are rated on a 5-point Likert scale (1 = *Never*, 5 = *Very Often*) and refer to gaming-related activities over the past 12 months. The scale is designed to capture symptoms of gaming disorder in both online and offline gaming contexts. Previous studies have reported Cronbach’s α values ranging from .81 to .96 across different languages and populations (39). In the present study, internal consistency was α = .78.

##### Brief aggression questionnaire

The Brief Aggression Questionnaire (BAQ) (40) is a 12-item self-report instrument measuring trait aggression across four subscales (Physical Aggression, Verbal Aggression, Anger, Hostility). Items are rated on a 7-point Likert scale (1 = *Extremely Uncharacteristic of Me*, 7 = *Extremely Characteristic of Me*). Previous studies reported Cronbach’s α values ranging from .79 to .83 (40). In the present study, internal consistency was α = .70.

##### Patient health questionnaire-9

Patient Health Questionnaire-9 (PHQ-9) (41) is a 9-item self-report instrument assessing depressive symptoms over the past two weeks. Items are rated on a 4-point Likert scale (0 = *Not at all*, 3 = *Nearly every day*). Previous studies reported Cronbach’s α values ranging from .86 to .89 (41). In the present study, internal consistency was α = .87.

##### Generalized anxiety disorder 7-item

Generalized Anxiety Disorder 7-item (GAD-7) (42) is a 7-item self-report instrument assessing generalized anxiety symptoms over the past two weeks. Items are rated on a 4-point Likert scale (0 = *Not at all*, 3 = *Nearly every day*). The original version demonstrated good reliability, with Cronbach’s alpha of .92 (42). In the present study, internal consistency was α = .89.

##### Gaming involvement questionnaire

Gaming time was measured using the Gaming Involvement Questionnaire (43), in which participants reported their average time spent on gaming and other gaming-related activities (e.g., watching gameplay, exploring game lore) on a typical weekday and weekend day. The average weekly gaming time was calculated by the sum of hours spent on gaming during average weekdays and average weekend days. In the present study, the tool was included primarily to verify whether participants could be classified as active gamers and to identify potential outliers with exceptionally high gaming times.

#### Statistical analysis

Basic statistical analyses (descriptive statistics, correlations) and exploratory factor analysis were conducted using IBM SPSS Statistics 25. Confirmatory factor analysis was performed in IBM SPSS AMOS 29 Graphics.

## Results

### Validation of rage in gaming questionnaire

To assess the psychometric properties of the Rage in Gaming Questionnaire (RIG), an Exploratory Factor Analysis (EFA) followed by a Confirmatory Factor Analysis (CFA) was conducted to verify the theoretical assumptions regarding its three subscales: Emotion, Cognition, and Arousal. The dataset was randomly split into two subsamples, with 129 observations used for EFA and 138 observations used for CFA. This approach was adopted to avoid overfitting and to ensure that the factor structure identified in the EFA could be independently validated in a separate sample using CFA (44). Additionally, to verify discriminant validity, correlations between RIG and other measures (IGDS9-SF, BAQ, DERS, PHQ-9, and GAD-7) were calculated.

### Exploratory factor analysis

An exploratory factor analysis (EFA) using the principal axis factoring method was conducted separately on Emotion, Cognition, and Arousal, with the number of factors set to one in each analysis. The analysis confirmed the adequacy of variable selection for EFA. The KMO values exceeded the commonly accepted threshold of 0.50, indicating sufficient intercorrelations among items and justifying the use of factor analysis. Bartlett’s test of sphericity was statistically significant for each subscale, indicating sufficient correlations between items to justify extracting a single factor. Results for each subscale are presented in **Table 2**. Based on communalities, items with low values (relative to their subscale) after factor extraction were excluded: “I experience mood swings” (.40), “I feel embittered” (31), “I feel stimulated” (.21), It’s harder for me to think” (.19), “I feel confused” (.19), “I lose focus in the game” (.12). These items were removed from further analyses.

**Table 2:** KMO, Bartlett’s test, and explained variance for each RIG subscale (EFA).

Subscale	KMO	Bartlett’s Test of Sphericity	df	% variance
Emotion	.90	429.90***	21	53.50%
Arousal	.73	124.50***	6	44.85%
Cognition	.74	165.33***	21	28.30%

***p <.001.

### Confirmatory factor analysis

**Figure 1:**
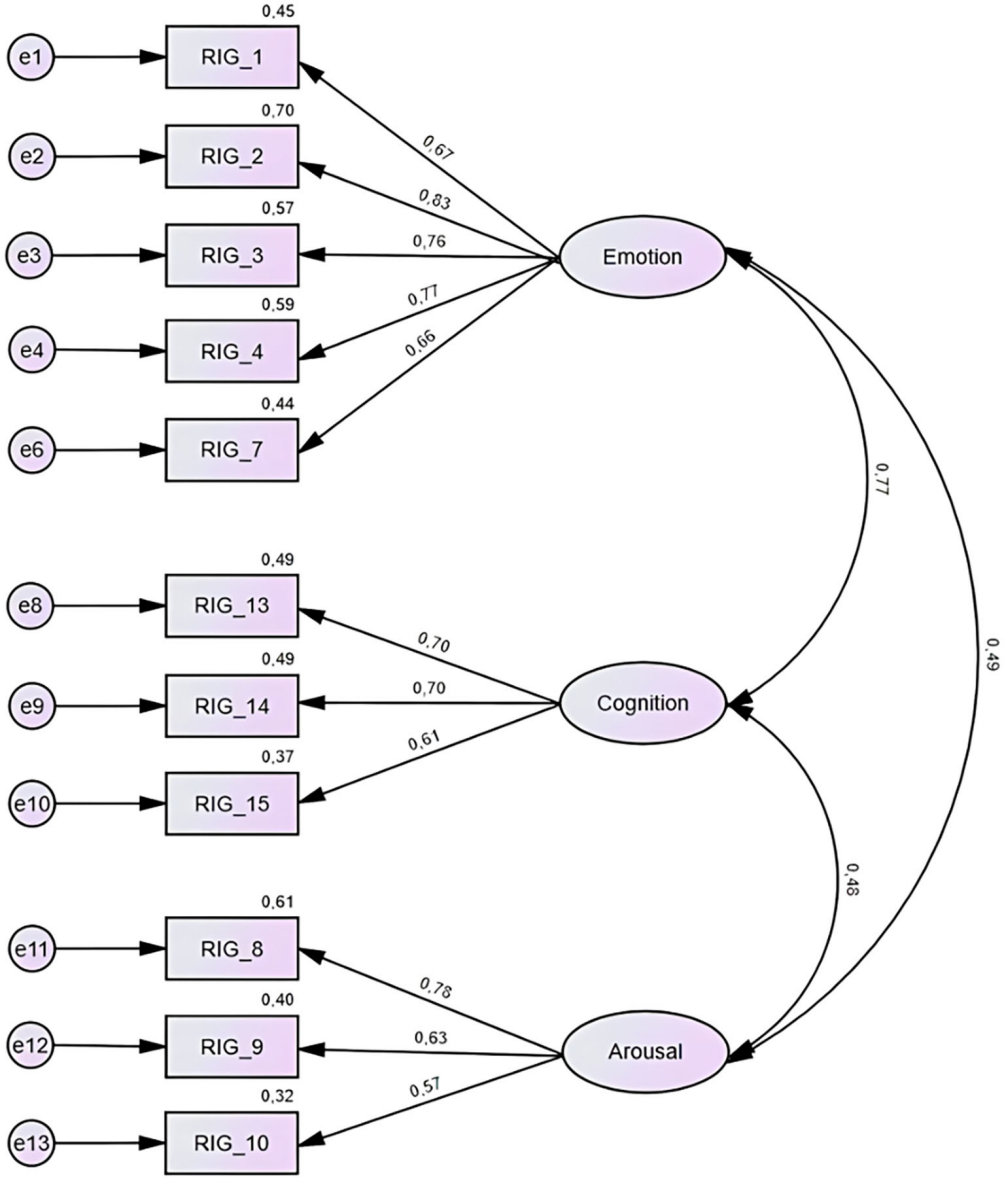
Final CFA model with three latent variables: Emotion, Cognition, and Arousal.

A Confirmatory Factor Analysis was conducted on the remaining 12 items. A factor loading cut-off value of .50 was applied, meaning that items loading below .50 on their respective factors were considered for removal. Based on this criterion, one item from the Cognition subscale (“I make impulsive decisions”) was excluded due to a factor loading slightly below the accepted threshold (.49). This decision was also supported by earlier concerns regarding whether the item reflects a core component of the construct or rather a behavioral outcome of tilt. No model modifications based on modification indices were applied, and the model was tested as specified by the theoretical framework. The final model, as illustrated in the **Figure 1**, included three latent variables. The final model demonstrated a good fit to the data: CMIN/DF = 1.316, CFI = .975, RMSEA = .048, and SRMR = .048, all of which meet the recommended international cut-off criteria (CFI ≥.95, RMSEA ≤.06, SRMR ≤.08; (45)). **Table 3** presents the final set of shortened items assigned to each of the three subscales: Emotion, Cognition, and Arousal, along with their respective factor loadings. The full wording of the items is available in the **Supplementary Materials**.

**Table 3:** Final structure and items of the Rage in Gaming Scale (RIG).

Subscale	Items	Factor loadings
Emotion	I feel annoyed	.67
	I feel irritated	.83
	I feel angry	.76
	I feel frustrated	.77
	I get upset more easily	.66
Arousal	I feel tension in my body	.78
	I feel as if my heart is beating faster	.63
	I notice involuntary bodily reactions (e.g., changes in breathing, sweating, hand shaking, or trembling	.57
Cognition	I feel powerless	.70
	I feel helpless	.70
	I feel disappointed	.61

### Internal consistency

Once the factor structure was determined, reliability of all three RIG scales was assessed. **Table 4** presents Cronbach’s alpha, Composite Reliability (CR), MacDonald’s Omega and Average Variance Extracted (AVE) statistics. The values were reported both for overall RIG scale and for three subscales. In the lower part of the table the diagonal shows square root AVE of a given subscale in brackets. The lower triangle of last three columns presents correlations between the subscales. The correlations between the subscales were statistically significant (*p* <.01) and moderate. In the context of the Fornell-Larcker criterion, the AVE roots for each subscale (values in diagonal brackets) were higher than their correlations with the other subscales, indicating satisfactory discriminant validity between the subscales and confirming that they measure distinct, yet related, aspects of anger in games.

**Table 4:** Reliability of Rage in Gaming (RIG).

Subscale	CR	ω	α	AVE	RIG	Emotion	Arousal	Cognition
RIG	.91	.86	.87	.49	(.70)			
Emotion	.86	.87	.87	.55	.91**	(.74)		
Arousal	.70	.74	.74	.44	.71**	.44**	(.67)	
Cognition	.71	.70	.68	.45	.77**	.62**	.34**	(.67)

CR, composite reliability; ω, McDonald’s omega; α, Cronbach’s Alpha; AVE, Average Variance Extracted.

**p <.01.

### Discriminant validity

To assess the discriminant validity of the Rage in Gaming Scale (RIG), correlations were calculated between RIG scores and measures of distinct psychological constructs. Additionally, to explore the relationship between rage, gaming disorder, and emotional dysregulation, correlations with relevant scales were also examined. As expected, all correlations were statistically significant. All results are presented in **Table 5**.

**Table 5:** Correlations among the variables.

Variables	*M*	*SD*	1	2	3	4	5	6
1. RIG	25.82	7.31	1					
2. IGD	17.48	5.31	0.42**	1				
3. BAQ	36.43	9.96	0.40**	0.17**	1			
4. DERS	42.75	12.23	0.30**	0.38**	0.33**	1		
5. PHQ	17.85	6.05	0.22**	0.35**	0.30**	0.62**	1	
6. GAD	14.27	5.24	0.30**	0.34**	0.28**	0.63**	0.80**	1

**p < 0,01.

## Discussion

The main aim of this paper was to develop a comprehensive definition and a novel measurement tool for the phenomenon of rage among video game players. Moreover, our purpose was to explore the relationships between rage, gaming disorder, and emotional dysregulation.

This study introduces a new questionnaire designed to assess rage as an emotional response in gamers, characterized by a deviation from their typical style of gameplay. Although a previously developed scale exists to assess rage in gaming contexts (12), it primarily focuses on behavioral manifestations of it, emphasizing its causes and consequences. The new tool was developed to deepen understanding of rage as an emotional phenomenon. It conceptualizes rage as an emotional reaction to in-game failure or frustration (e.g., losing, negative social interactions), expressed on emotional, arousal, and cognitive levels, ultimately impacting behavior. This scale complements rather than replaces the existing one and used together they enable a more comprehensive assessment of both the emotional core of rage and its behavioral outcomes.

The scale was developed based on the GAM (30), prior research on rage in gaming, and findings from our qualitative study. This multi-source approach ensured both theoretical grounding and relevance to players’ lived experiences. Importantly, the RIG directly operationalizes the internal states component of GAM, capturing emotion, cognition, and arousal as the immediate processes through which rage manifests in gaming contexts. This allows researchers to examine rage not only as an isolated affective reaction but as a dynamic mechanism embedded in the broader GAM framework. While input factors and decision processes, were not directly measured in the present study, the RIG provides a foundation for exploring how these elements interact with internal states in future research. For example, the tool enables testing whether trait impulsivity or exposure to permissive attitudes toward aggression amplify the intensity of rage, and how regulatory skills shape whether this state translates into verbal aggression or rage-quitting. The universality of GAM makes it particularly well suited for interactive gaming environments, where player reactions result from the interplay of personality dispositions, temperamental factors, group dynamics, and situational frustrations. By embedding rage within this broader theoretical model, the RIG contributes to a more comprehensive understanding of how emotional states emerge and unfold in digital play.

To empirically verify the theoretical structure of the scale, both exploratory factor analysis (EFA) and confirmatory factor analysis (CFA) were conducted sequentially. During the EFA stage, the Kaiser-Meyer-Olkin (KMO) value was well above the recommended threshold of 0.5, indicating the suitability of the data for factor analysis. Seven items were removed due to low factor loadings after extraction. In the CFA stage, the item “I make impulsive decisions” was excluded due to a factor loading slightly below the .50 threshold and prior concerns from expert that it may reflect a consequence rather than the core experience of rage, which were supported by the statistical results. Additionally, a conceptually similar item “I make decisions without thinking” is included in the scale developed by Bonilla and colleagues (12), further supporting the idea that the two scales may be treated as complementary rather than redundant. The final model showed a very good fit to the data.

The developed tool includes three subscales: Emotion (α = .87), Cognition (α = .68), and Arousal (α = .74), all demonstrating satisfactory reliability. The overall scale also showed good internal consistency (α = .86). The lowest alpha value was observed for the Cognition subscale, which was marginally below the commonly accepted .70 threshold (46). However, the reliability of this subscale can be regarded as sufficient given the limited number of items, as Cronbach’s alpha is known to be influenced by item count (47), with fewer items typically yielding lower alpha values. Nonetheless, some limitations of this subscale should be noted. It accounted for only 28.3% of the variance in the exploratory factor analysis and showed a suboptimal average variance extracted (AVE = .45), suggesting that it may not fully capture the breadth of the intended construct and could benefit from further refinement to improve conceptual clarity. Notably, McDonald’s omega values were slightly higher than Cronbach’s alpha across most subscales, which is consistent with the known property of ω being less sensitive to the number of items and more robust when item loadings are not uniform. Composite reliability (CR) was high for the overall scale (CR = .91) and the Emotion subscale (CR = .86), indicating that a substantial proportion of the variance in scores can be attributed to the underlying latent constructs. Although the AVE values for Cognition and Arousal were slightly suboptimal, their convergent validity may still be considered acceptable in light of theoretical alignment and overall model fit. Future iterations of the scale may benefit from expanding or redefining the Cognition domain to improve both psychometric robustness and conceptual clarity.

A moderate positive correlation between rage and gaming disorder symptoms was found, reflecting a potential relationship between the two constructs while preserving their distinctiveness – each addressing a different aspect of gaming. Although this association is statistically significant, its magnitude remains modest, and interpretations should be made with caution. This preliminary finding shows that rage during gameplay is associated with problematic gaming behaviors, but further research is needed to clarify its role. A statistically significant medium-sized correlation was found between rage and trait aggression, reflecting some overlap between the tendency to experience rage during gameplay and a general disposition toward aggressive behavior. However, the constructs remain distinct, and further research is needed to better understand their relationship. Furthermore, a statistically significant small-sized association between rage and emotional dysregulation provides additional support for the emotional basis of rage in gaming contexts, showing that individuals who struggle to regulate their emotions may be more prone to experiencing rage during gameplay. Small but significant correlations were also observed between rage and depressive symptoms as well as generalized anxiety symptoms. These results may reflect a broader link between gaming-related rage and emotional difficulties, though the strength of these associations warrants cautious interpretation. Taken together, these findings should be considered exploratory and hypothesis-generating. Future research, particularly using regression or longitudinal designs, is needed to better understand the directionality and potential mediating or moderating mechanisms in these relationships.

### Limitations

Several measurement-related limitations should be considered when interpreting the findings of this study. As all measures were self-reports administered in a single session, the results may be affected by common method bias and self-report distortions, such as tendencies toward social desirability. The absence of behavioral or physiological validation further constrains the conclusions, as future studies could include objective indicators such as observed in-game behaviors (e.g., use of profanity) or psychophysiological measures (e.g., heart rate variability, electrodermal activity).

In addition, the study did not assess test–retest reliability and relied on a single time-point measurement, which limits the ability to evaluate the temporal stability of the construct. The lack of measures assessing impulsivity and ADHD further constrains the construct validity of the scale. Both constructs, like rage, involve impulsive responses and heightened sensitivity to stimuli; thus, it is important to determine whether the scale can effectively differentiate between these related constructs. Moreover, as impulsivity (48) and ADHD (49) have been strongly linked to gaming disorder, including these variables in future validation studies would clarify discriminant validity and provide a more nuanced understanding of rage in gaming contexts, as individuals with elevated impulsivity or ADHD symptoms may be more prone to rage reactions.

Another limitation concerns the omission of an existing measure of gaming rage – the Tilt Scale (12) which focuses on the behavioral dimension of rage. Although the present scale adopts a distinct theoretical approach by conceptualizing rage primarily as an emotional experience, comparing both instruments could offer valuable insights. While RIG emphasizes the affective and cognitive underpinnings of rage episodes, TILT captures situational triggers and behavioral outcomes, making the two tools potentially complementary. For example, RIG may be particularly useful in studies linking rage with mental health indicators, while TILT may be more applicable in research on performance decline, particularly in the context of esports. Using both measures in future research may therefore provide a more comprehensive understanding of rage phenomena across emotional and behavioral dimensions. Including the existing scale in future studies would allow for a direct comparison of results and contribute to a clearer delineation of the construct.

Several sample-related limitations should be acknowledged. The qualitative focus group included only four participants, which inevitably restricted the breadth of rage experiences captured. Although qualitative research prioritizes the adequacy and richness of data rather than strict numerical criteria (50), this small group should be regarded as providing preliminary insights. Future studies should therefore include larger and more diverse samples to enhance the representativeness and comprehensiveness of qualitative findings.

Another limitation of the study concerns the sample size in the quantitative phase. There is no consensus in the literature regarding the required sample size for factor analysis. While rules of thumb e.g., 10 participants per item (51) are common, they are often criticized as inadequate. More structured guidelines suggest sample sizes from 100 to 500, depending on variables and factor loadings (52). In this study, the sample was split for EFA (N = 129) and CFA (N = 138). Despite adequate factor loadings (>.50) and a limited number of items and factors, the relatively modest sample may still restrict the generalizability of the results.

Additionally, because the sample was self-selected and recruited online, it may not fully represent the broader gaming population. The absence of cross-cultural validation further limits the external validity and generalizability of the findings. Finally, given the modest sample size, the study may have been underpowered to detect small effects; therefore, the results should be interpreted with caution.

Although several external measures were included, the present study relied solely on correlations to examine external validity. Because multiple correlations were conducted without correction for multiple comparisons, the results should be interpreted with caution. As this is a preliminary validation, future research should employ more advanced statistical approaches, such as regression analyses, structural equation modeling, and measurement invariance testing, together with more stringent correction methods to provide stronger validity evidence.

Finally, the cross-sectional nature of the study limits the conclusions that can be drawn, as causal inferences cannot be established. Longitudinal designs are therefore needed in future research to examine temporal dynamics and provide stronger validity evidence regarding the directionality and stability of the observed relationships.

### Further directions

The rage scale developed in this study should be regarded as a preliminary research tool with promising potential for capturing rage in gaming contexts. Further validation is needed, particularly in light of the study’s limitations, and future research may also include intervention studies to explore the scale’s practical utility in clinical and educational contexts. At this stage, however, the RIG is intended solely for research purposes; subsequent studies will be required to establish normative data, preliminary score interpretation guidelines, and potential cutoff points before the scale can be considered for screening or monitoring. Future research should also examine its applicability across different gaming genres and populations, while clearly defining its appropriate scope of use to prevent misuse.

Future research should aim to replicate the findings using larger and more diverse samples, including clinical populations and adolescents. Adolescents, due to their developmental stage and increased emotional reactivity (53), may be especially prone to experiencing rage during gameplay, making them a particularly relevant group for further investigation. To establish causal relationships and assess the stability of rage experiences over time, longitudinal research designs are recommended. Moreover, future studies should examine potential cross-cultural differences in the experience and expression of rage, as emotional responses and their regulation are known to be shaped by cultural norms and display rules (54). Identifying such differences may help clarify the generalizability of the construct and inform culturally sensitive assessment and intervention strategies. A useful direction for future research would be to incorporate descriptive comparisons, such as by gender or gaming hours, to better situate the observed results. Finally, behavioral validation studies, for instance through the observation of in-game aggressive behaviors, could provide complementary evidence beyond self-report and contribute to a more comprehensive validation of the scale.

A promising direction is exploring the relationship between rage and player motivations, as defined by the Gaming Motivation Inventory (GMI) (55). In particular, competitive motivation may be closely linked to rage, and has been identified as a significant predictor of gaming disorder (55). Investigating this connection could provide insight into how in-game goals and emotional reactions interact. This relationship may be particularly relevant in competitive genres such as MOBA or FPS games, where performance pressure and social evaluation intensify emotional responses and increase the likelihood of rage (15). Moreover, although gaming time alone is considered a weak predictor of gaming disorder (56), future research should examine its interaction with rage, as rage may serve as an additional variable helping to explain the relationship between playtime and problematic gaming.

Additionally, the role of alexithymia in rage should be further explored, as alexithymia is also a known predictor of gaming disorder (57). Some individuals with alexithymia might use video games as a safe environment to externalize or release negative emotions they struggle to process otherwise. This raises the possibility that rage in gaming contexts could function as a unique emotional outlet for those with impaired emotional awareness, highlighting the need to better understand its psychological underpinnings. A promising direction for future research is the examination of rage in a social context, particularly its impact on the emotions and behaviors of other players. It would be valuable to explore how one player’s expression of rage influences team dynamics, cooperation, and the emotional experiences of their teammates. Altogether, these directions may eventually contribute to a more nuanced understanding of rage in gaming contexts and, in the long term, inform its possible clinical applications.

## Data Availability

The raw data supporting the conclusions of this article will be made available by the authors, without undue reservation.
