# Supplementary material for: The anatomy of rage: conceptualization, operationalization, and validation of a new measurement scale
Source: Front Psychiatry. 2025 Nov 17;16:1648903. doi: 10.3389/fpsyt.2025.1648903 (PMC12667240; doi:10.3389/fpsyt.2025.1648903)
Supplement: Supplementary file 1 [file DataSheet1.pdf]

Supplementary Materials

Description of Focus Group Participants and Example Quotations

Participant A – Female, 23 years old, university student.
Self-reported rage level: 2.
Preferred genre: unspecified, generally single-player story-driven games.
Primary gaming motivation: relaxation, distraction from problems, and mental engagement.

Once, during a rage episode, I didn't feel like myself at all — it was as if someone else had taken over me. (Participant A)

Participant B – Female, 22 years old, university student.
Self-reported rage level: 3.
Preferred genre: cozy games or story-driven games.
Primary gaming motivation: entertainment and escape from reality.

Rage feels like a building pressure, like water in a pot that's about to boil over. (Participant B)

Participant C – Male, 28 years old, employed.
Self-reported rage level: 4.
Preferred genre: RPG and MOBA.
Primary gaming motivation: relaxation, immersion in fictional worlds, and skill improvement.

During rage, I feel disappointed in myself, like I didn't show what I'm truly capable of. (Participant C)

Participant D – Male, 44 years old, employed.
Self-reported rage level: 6.
Preferred genre: FPS and MMORPG.
Primary gaming motivation: competition, skill mastery, and detachment from the outside world.

It's a sense of helplessness — like there's nothing more I can do. (Participant D)

RIG – Rage in Gaming preliminary set of items

1. I feel frustrated
2. I feel stressed
3. I feel annoyed
4. I feel irritated
5. I feel angry
6. I feel upset
7. I feel nervous
8. I feel sad
9. I feel disappointed
10. I feel powerless
11. I feel helpless
12. I feel strong emotional tension
13. I feel tension in my body
14. I feel as if my heart is beating faster
15. I feel that it’s harder for me to breathe
16. I feel that I’m sweating more
17. I feel embittered
18. I notice involuntary bodily reactions (e.g., changes in breathing, sweating, hand shaking, or trembling)
19. I feel that my hands shake or tremble
20. I feel stimulated
21. I feel that it’s my fault
22. I feel I get upset easier than normal
23. I have negative thoughts
24. I lose focus in the game
25. I have trouble concentrating
26. I have trouble planning further actions and making reasonable decisions
27. I don't recognize myself
28. I don’t follow the strategy I previously planned
29. I am confused
30. I feel lost
31. It’s harder for me to think
32. I make impulsive decisions
33. I blame myself
34. I blame other players
35. I blame the game
36. I feel like I have no control over myself
37. I am hard on myself
38. I have trouble remembering necessary information
39. I don't think about the consequences of my actions
40. I experience mood swings

Table 1

Correlation matrix of individual RIG items

Item	*M*	*SD*	Skew	Kurt	1	2	3	4	5	6	7	8	9	10	11
1. I feel annoyed	2.67	0.98	0.61	-0.44											
2. I feel irritated	2.69	1.03	0.19	-0.43	.59**										
3. I feel angry	2.31	0.97	0.63	0.22	.54**	.64**									
4. I feel frustrated	2.69	0.98	0.19	-0.50	.64**	.64**	.62**								
5. I get upset more easily	2.40	1.06	0.50	-0.35	.50**	.55**	.56**	.52**							
6. I feel powerless	2.04	0.94	0.59	-0.41	.41**	.46**	.40**	.42**	.39**						
7. I feel helpless	2.05	0.99	0.85	0.38	.37**	.41**	.37**	.46**	.28**	.54**					
8. I feel disappointed	2.46	0.96	0.22	-0.32	.40**	.42**	.39**	.46**	.31**	.41**	.31**				
9. I feel tension in my body	2.30	1.05	0.40	-0.62	.30**	.32**	.28**	.32**	.30**	.27**	.20**	.22**			
10. I feel as if my heart is beating faster	2.22	1.09	0.52	-0.62	.32**	.32**	.30**	.28**	.30**	.15*	.15*	.32**	.45**		
11. I notice involuntary bodily reactions (e.g., changes in breathing, sweating, hand shaking, or trembling)	1.97	1.10	0.98	0.12	.33**	.29**	.23**	.21**	.29**	.21**	.21**	.20**	.52**	.48**	

Note. Skew = skewness; Kurt = kurtosis.
*$p < .05$
**$p < .01$

Table 2
Item retention and removal process

Item	1	2	3	4	5	Deletion reason
I feel nervous	8	YES	-	-	-	Expert rating of 1 and low average score.
I feel sad	7	YES	-	-	-	
I feel that it’s harder for me to breathe	7	YES	-	-	-	
I feel that I’m sweating more	9	YES	-	-	-	
I feel that it’s my fault	6	YES	-	-	-	
I have negative thoughts	8	YES	-	-	-	
I have trouble concentrating	8	YES	-	-	-	
I have trouble planning further actions and making reasonable decisions	9	YES	-	-	-	
I don't recognize myself	5	YES	-	-	-	
I feel like I have no control over myself	9	YES	-	-	-	
I feel lost	7	YES	-	-	-	
I am hard on myself	7	YES	-	-	-	
I have trouble remembering necessary information	6	YES	-	-	-	
I don't think about the consequences of my actions	9	YES	-	-	-	
I don’t follow the strategy I previously planned	9	YES	-	-	-	
I blame other players	9	YES	YES	-	-	Expert rating of 1 and low average score, subscale mismatch.
I feel strong emotional tension	11	-	YES	-	-	Subscale mismatch.
I feel that my hands shake or tremble	12	-	-	-	-	Item redundancy.
I don’t follow the strategy I previously planned	9	YES	-	-	-	Expert rating of 1 and low average.
I blame the game	11	-	YES	-	-	Subscale mismatch.
I feel embittered	12	-	-	.31	-	Low values after factor extraction compared with other subscale items.
I feel stimulated	13	-	-	.21	-	
I lose focus in the game	12	-	-	.12	-	
I am confused	11	-	-	.19	-	
It’s harder for me to think	10	-	-	.19	-	
I experience mood swings	11	-	-	.40	-	
I make impulsive decisions	11	-	YES	.44	.49	Below loading threshold and reflected behaviour rather than emotional core.
I feel frustrated	13	-	--	.67	.77	Retained.
I feel annoyed	14	-	-	.63	.67	
I feel irritated	14	-	-	.65	.83	
I feel angry	13	-	-	.61	.76	
I feel disappointed	12	-	-	.30	.61	
I feel powerless	12	-	-	.43	.70	
I feel helpless	11	-	-	.32	.70	
I feel tension in my body	12	-	-	.47	.78	
I feel as if my heart is beating faster	11	-	-	.48	.63	
I notice involuntary bodily reactions (e.g., changes in breathing, sweating, hand shaking, or trembling)	9	-	-	.64	.57	
I feel I get upset easier than normal	11	-	-	.49	.66	

Note.
1 = Expert ratings; 2 = Expert rating of 1; 3 = Assigned to multiple subscales; 4 = Values after factor extraction; 5 = Factor loadings.

RIG – Rage in Gaming

Authors: Michałkiewicz, N., Strojny P., Strojny, A.

Sometimes, while playing, you may experience **changes** in **emotions, thoughts, or feelings** that **differ from your typical way of playing**.

Below, you will find several statements - please rate how often you experience such changes.

For example, if you **always feel joy** while playing, **do not** consider this **a change.**

Legend: 1 = Never - I do not notice such a change, 2 = Rarely, 3 = Sometimes, 4 = Often, 5 = Very often - I notice such a change

	1	2	3	4	5
How often do you **feel annoyed** compared to your usual way of playing?					
How often do you **feel irritated** compared to your usual way of playing?					
How often do you **feel angry** compared to your usual way of playing?					
How often do you **feel frustrated** compared to your usual way of playing?					
How often do you **get upset more easily than normal** compared to your usual way of playing?					
How often do you **feel powerless** compared to your usual way of playing?					
How often do you **feel helpless** compared to your usual way of playing?					
How often do you **feel disappointed** compared to your usual way of playing?					
How often do you **feel tension in your body compared** to your usual way of playing?					
How often does your **heart feel like it is beating faster** compared to your usual way of playing?					
How often do you **notice involuntary bodily reactions (e.g., changes in breathing, sweating, hand shaking, or trembling)** compared to your usual way of playing?					
